# Economic Evaluation of Text-Messaging and Smartphone-Based Interventions to Improve Medication Adherence in Adolescents with Chronic Health Conditions: A Systematic Review

**DOI:** 10.2196/mhealth.6425

**Published:** 2016-10-25

**Authors:** Sherif M Badawy, Lisa M Kuhns

**Affiliations:** ^1^ Ann and Robert H Lurie Children's Hospital of Chicago Department of Pediatrics, Division of Hematology, Oncology, and Stem Cell Transplant Northwestern University Feinberg School of Medicine Chicago, IL United States; ^2^ Faculty of Medicine Department of Pediatrics, Division of Hematology and Oncology Zagazig University Zagazig Egypt; ^3^ Ann and Robert H Lurie Children's Hospital of Chicago Department of Pediatrics, Division of Adolescent Medicine Northwestern University Feinberg School of Medicine Chicago, IL United States

**Keywords:** adolescent, text messaging, smartphone, medication adherence, chronic disease, cost-benefit analysis

## Abstract

**Background:**

The rate of chronic health conditions (CHCs) in children and adolescents has doubled in the past 20 years, with increased health care costs. Technology-based interventions have demonstrated efficacy to improving medication adherence. However, data to support the cost effectiveness of these interventions are lacking.

**Objective:**

The objective of this study is to conduct an economic evaluation of text-messaging and smartphone-based interventions that focus on improving medication adherence in adolescents with CHCs.

**Methods:**

Searches included PubMed MEDLINE, Embase, Cochrane Central Register of Controlled Trials, Cumulative Index to Nursing and Allied Health Literature, PsycINFO, Web of Science, and Inspec. Eligibility criteria included age (12-24 years old), original articles, outcomes for medication adherence, and economic outcomes.

**Results:**

Our search identified 1118 unique articles that were independently screened. A total of 156 articles met inclusion criteria and were then examined independently with full-text review. A total of 15 articles met most criteria but lacked economic outcomes such as cost effectiveness or cost-utility data. No articles met all predefined criteria to be included for final review. Only 4 articles (text messaging [n=3], electronic directly observed therapy [n=1]) described interventions with possible future cost-saving but no formal economic evaluation.

**Conclusions:**

The evidence to support the cost effectiveness of text-messaging and smartphone-based interventions in improving medication adherence in adolescents with CHCs is insufficient. This lack of research highlights the need for comprehensive economic evaluation of such interventions to better understand their role in cost-savings while improving medication adherence and health outcomes. Economic evaluation of technology-based interventions can contribute to more evidence-based assessment of the scalability, sustainability, and benefits of broader investment of such technology tools in adolescents with CHCs.

## Introduction

The rate of chronic health conditions (CHCs) in children and adolescents (eg, asthma, diabetes) has doubled in the past 20 years [1,2]. Adolescents (12-24 years old) with CHCs, a special subpopulation of pediatric patients, face the day-to-day challenges of transitioning to adult responsibilities while simultaneously managing their illness-related routine [3-5]. Adolescence is an important time to develop healthy habits and behaviors, and building self-management skills is a critical component of successful transition to adulthood. Engaging adolescents with CHCs in self-management skill building is an invaluable investment with long-term benefits. In particular, medication adherence is a crucial component of self-management, and poor adherence is a common problem in adolescents with CHCs [3]. Across pediatric chronic conditions, this can have negative effects on morbidity, mortality, and quality of life with increased use of health services and annual health care cost [3,6-10].

Taking daily medications is a challenge irrespective of the frequency, formulation, or patient’s age. There are possible differences in adherence barriers across chronic conditions, such as disease-specific treatment regimens and monitoring requirements. However, evidence from a recent systematic review suggests that among adolescents with chronic conditions, most perceived barriers are not unique to a specific disease state [11]. Nevertheless, barriers to medication adherence among adolescents may be multifaceted, and there may be common attributes to this phenomenon that could be amenable to interventions across chronic conditions.

Adolescents have adopted communication technology such as cellular phones, the Internet, and social networking at a rapid rate across levels of social position and status [12-14]. Recent developments in information and communications technologies have opened new opportunities to improve health care and to link patients and their providers. A recent report from a Pew Internet research survey found that teens have access to smartphones, tablets, desktop computers, and laptop computers at rates of 73%, 58%, 87%, and 81%, respectively [12,13]. This presents an opportunity to promote self-management and medication adherence among adolescents via these technologies. The use of portable and easily accessible technology-based interventions, particularly text messaging and smartphone apps, to address health-related problems has been shown to be both feasible and acceptable for different health conditions [15-17]. In addition, previous systematic reviews and meta-analyses of pediatric patients with CHCs have shown considerable positive effects of these types of interventions in improving medication adherence, health-related quality of life (HRQOL), and family functioning [15-21]. However, the cost effectiveness of developing and maintaining such technology-based interventions remains poorly understood.

The effect of technology-based interventions on health care costs in adolescents with CHCs may go beyond the direct cost savings associated with medication adherence and related health outcomes. These interventions may facilitate efficient health care operations (eg, fewer missed clinic, screening, or laboratory monitoring appointments), increased access to high-quality care (eg, timely referrals to other services for consultations), and potential cost savings of labor. Therefore, economic evaluation of technology-based interventions can contribute to a better understanding of the scalability, sustainability, and benefits of broader investment of such technology tools. Economic data may also raise considerations for third-party reimbursement should interventions prove to be effective in improving health outcomes in this population. The objectives of this systematic review are to (1) conduct an economic evaluation (cost effectiveness and cost-utility analyses) of text-messaging and smartphone-based interventions that focus on improving medication adherence in adolescents with CHCs and (2) determine whether the incremental benefit gained from using such interventions is enough to justify the additional cost required to adopt, develop, and maintain the intervention.

## Methods

### Search Strategy

The authors collaborated with a librarian who developed the search strategies and from July to September 2015 ran searches in the following databases: PubMed MEDLINE, Embase (embase.com), Cochrane Central Register of Controlled Trials (CENTRAL) on the Wiley platform, Cumulative Index to Nursing and Allied Health Literature (CINAHL) (EBSCO), PsycINFO (EBSCO), Web of Science, Center for Review and Dissemination (CRD); and Inspec (EBSCO). Further searches were run in November 2015 using the following sources: ProQuest dissertations, Scopus, ClinicalTrials.gov, World Health Organization clinical trials, Controlled-Trials.org, Institute for Electrical and Electronics Engineers (IEEE) Xplore, and Google Scholar. Search strategies for all databases except MEDLINE were adapted from the PubMed MEDLINE strategy. All databases were searched back to 1995 with no language limits applied. The search strategy looked for all articles on text messaging, phones, mobile apps, and portable software combined with adherence or compliance, and search terms related to child, pediatric, adolescent, and youth. Multimedia Appendix 1 shows the complete search strategies in each database. The authors also attempted to discover additional studies by searching the reference lists of key studies and relevant systematic reviews. We followed the guidelines for Preferred Reporting Items for Systematic Reviews and Meta-Analyses (PRISMA) in the report of evidence across the studies reviewed herein [22].

### Inclusion and Exclusion Criteria

The inclusion criteria were as follows: (1) adolescents (12-24 years old) with a CHC that requires long-term daily or weekly medications for 12 months or longer [23], (2) original research manuscripts, (3) studies that were either randomized controlled trials, quasi-experimental studies, or pilot/feasibility studies (including single arm, pretest/posttest), (4) text-messaging or smartphone-based interventions (app or mobile intervention), and (5) medication adherence as the primary or the secondary outcome. The exclusion criteria included the following: (1) mean or median age of participants younger than 12 years old or older than 24 years old or mean or median age not specified in the article, (2) adolescent participants not the focus of the study intervention (eg, interventions that target babies born to adolescent mothers with HIV or target parents of adolescent patients with CHCs), (3) interventions focused on disease monitoring or ecological momentary assessment but not designed to improve medication adherence, (4) technology-based interventions other than text messaging and smartphone apps, and (5) lack of economic outcomes such as cost effectiveness or cost-utility data.

### Data Extraction

We developed a standardized form for data extraction from the final included articles, adjusted for this particular study. Data items in the extraction form included the following: first author's name; publication year; country; CHC; participant ages; study design; duration of intervention and follow-up; components of technology intervention (text messaging or smartphone apps); adherence measures and rates; disease-related outcomes; theoretical framework; and economic outcomes including cost effectiveness and/or cost-utility data (eg, cost components of each intervention), incremental cost-effectiveness ratios (ICERs), quality adjusted life years (QALYs), and sensitivity analyses. We planned to evaluate the quality of evidence using the GRADE (Grades of Recommendation, Assessment, Development, and Evaluation) approach [24].

## Results

### Overview

**Figure 1:**
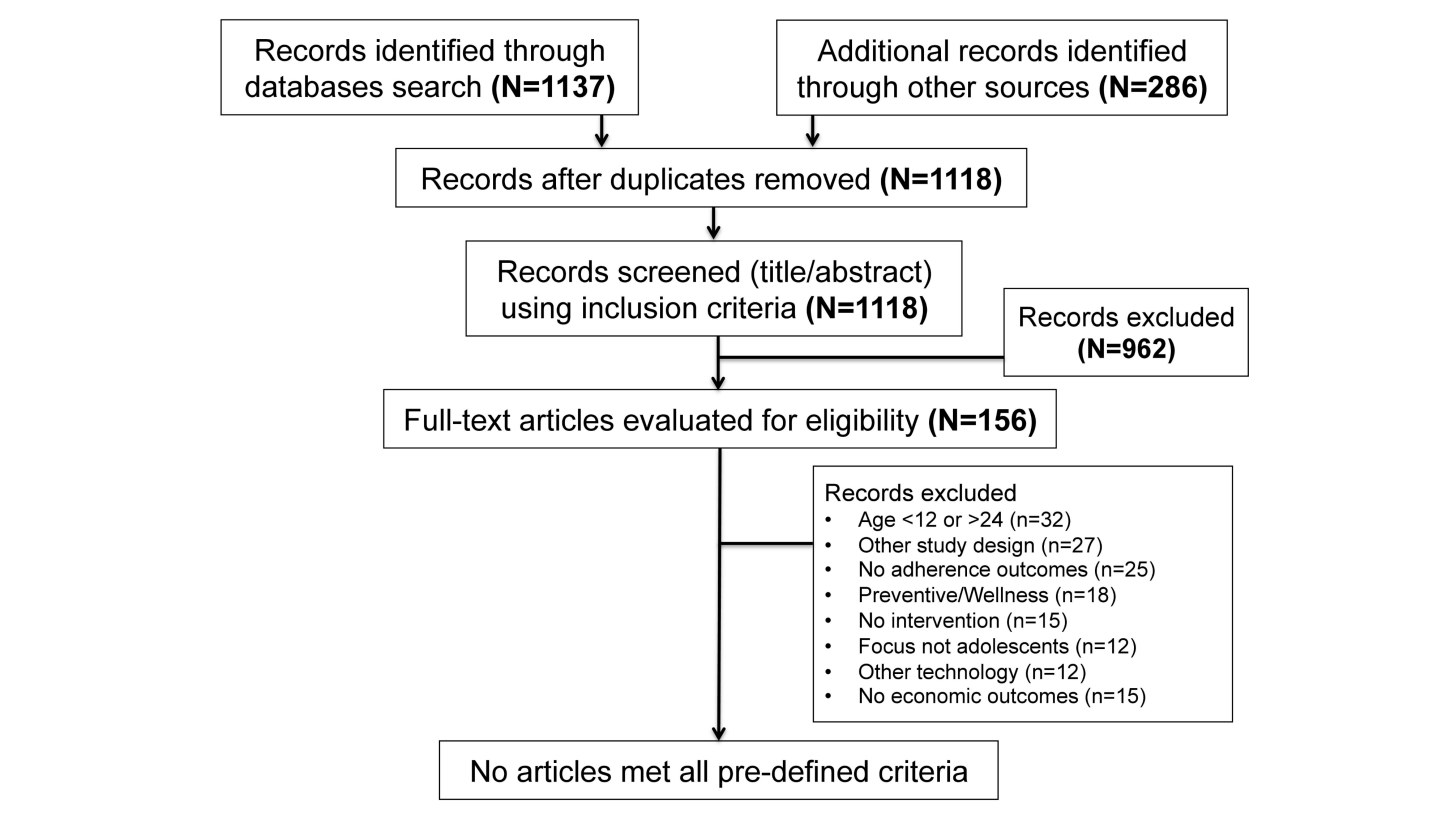
Flow of studies through the review according to the Preferred Reporting Items for Systematic Reviews and Meta-Analyses (PRISMA) guidelines.

The initial search retrieved 1137 records from the main electronic databases (PubMed, Embase, PsycINFO, CINAHL, CENTRAL, Web of Science, Inspec, CRD, and IEEE Xplore). We identified an additional 286 records from the gray literature and hand search of the bibliography of other systematic reviews. After removal of the duplicates, 1118 original articles remained (Figure 1). The authors independently screened the article titles and abstracts, and of those screened, 156 articles met the inclusion criteria. The authors then independently reviewed the full text of the 156 articles against the exclusion criteria. No articles met all predefined criteria to be included for final review. The reasons for exclusion of full text papers were documented in an adapted PRISMA study flowchart (Figure 1) [22]. It is worth noting that 15 articles met most predetermined inclusion and exclusion criteria but lacked economic outcomes such as cost effectiveness or cost-utility data. In addition, only 4 articles (text messaging [n=3] and mobile directly observed therapy [DOT] [n=1]) described interventions with possible future cost saving but no formal economic evaluation. Therefore, we summarized data from these 4 articles and suggested an economic evaluation approach for future studies.

### Study Characteristics

Creary et al were able to achieve a significant improvement in hydroxyurea adherence rates (93.3%) among children and adolescents with sickle cell disease by using mobile DOT [25]. The authors suggested that mobile DOT could be a cost-effective intervention as it has the potential for wider application with lower technology cost over time and better facial recognition capabilities [25]. The authors also projected that mobile DOT could decrease health care use because patients with higher adherence to hydroxyurea would have fewer disease-related complications, hospitalizations, and visits to the emergency department [25]. Another study by Ting et al using text message reminders showed statistically significant improvement in clinic attendance rates among adolescents with childhood-onset systemic lupus erythematosus but no improvement in adherence to their medication (hydroxychloroquine) [26]. However, the authors suggested that using a text-messaging approach might prove to be cost effective by reducing health care cost while improving clinic adherence and health outcomes overall [26]. Moreover, Miloh et al have shown that using a text-messaging approach not only significantly improved adherence to immune-suppressive medications in pediatric recipients of liver transplant but also reduced rejection episodes, suggesting possible cost-saving effects [27]. The authors further highlighted that the success of an implemented text-messaging approach lies in the characteristics of the intervention itself being personal, discreet, simple, socially acceptable, minimally intrusive, and low cost and requiring minimal time commitment from health care providers [27]. Furthermore, Franklin et al evaluated the efficacy of a text-messaging support system—Sweet Talk—among adolescents with diabetes mellitus type 1 [28]. The authors reported improvement in hemoglobin A_1c_ in patients who received intensive insulin therapy in addition to Sweet Talk and improvement in self-efficacy and adherence by self-report (in comparison to conventional therapy plus Sweet Talk) [28]. The authors highlighted that a text-messaging intervention may help to overcome critical limitations of the current more labor-intensive approach to diabetes education and management, such as cost and time commitment [28]. The authors also projected that text messaging might be a low-cost behavioral support intervention that can address the need for long-term behavior change and be integrated into routine care in the clinic setting with detailed cost-effectiveness evaluations [28].

### Suggested Approach for Economic Evaluation

Health economic evaluation of technology-based interventions (eg, text messaging and smartphone apps) helps to highlight the added value of these interventions by addressing two important points: (1) whether the technology-based intervention used to improve medication adherence among adolescents with chronic conditions improves health outcomes relative to other existing interventions and (2) whether the incremental benefit gained from using the technology-based intervention is enough to justify the additional cost required to adopt, develop, and maintain the intervention. Data on ICERs per unit improvement in medication adherence, disease-related outcomes, and QALYs would inform health economic evaluation and aid in the development of a cost-effectiveness model to evaluate whether these improvements are worth the costs required to develop, maintain, and disseminate the intervention. Disease-related outcomes include disease-specific complications, mortality, and HRQOL. In addition, evaluation of health and social care costs are important to consider in relation to HRQOL. Health economic evaluation should include a comprehensive cost analysis of the development, maintenance, and dissemination of technology-based interventions. A cost-effectiveness model using a cost-utility analysis of technology-based interventions for improving patient outcomes could be measured in terms of ICER per unit improvement in disease-related outcomes or QALY gained compared to standard of care. The ICER per extra QALY generated by text-messaging or smartphone app interventions to improve medication adherence over standard of care can be calculated using the following equations:

• (cost_text messaging_ - cost_standard of care_)/ (QALY_text messaging_ - QALY_standard of care_)

• (cost_smartphone app_ - cost_standard of care_)/ (QALY_smartphone app_ - QALY_standard of care_)

• (cost_smartphone app_ - cost_text messaging_)/ (QALY_smartphone app_ - QALY_text messaging_)

## Discussion

### Principal Findings

In our study, we were not able to identify any articles that met all our predefined criteria. Only 4 articles described interventions with possible future cost saving but no formal economic evaluation. Deriving QALY for use in cost-utility analysis in pediatric populations is a challenging task with only a few child-specific preference-based measures available. There is no single preference-based utility measure that has been validated for children of all ages in different health states, including adolescents with chronic conditions [29]. Time period is another important consideration in terms of evaluating short-term and, more commonly, long-term outcomes. Alternatives include using adult instruments, proxy measures, expert opinion, or published catalogs of pediatric utility values for different chronic conditions [29]. Individual preferences for health states can be elicited by either direct or indirect measures. Direct measures include standard gamble and time trade-off. The standard gamble is a technique to measure individuals' preferences under uncertainty and to express the outcome of different therapeutic choices in utility values that can be used in clinical decision analysis and health program evaluation. The time trade-off is another technique to elicit individuals’ preferences for health states by letting them imagine living a defined number of years in an imperfect health state then indicate the number of remaining life years in full health at which they are indifferent between the longer period of impaired health and the shorter period of full health. The challenge lies in adolescents’ ability to interpret some of those measures. They may lack the cognitive ability to understand the abstract concepts included in standard gambles or time trade-offs, especially the ones related to time spent in different health states and the possibility of death [30]. Chaining has been suggested as a technique to address these issues [31]. For example, the worst possible health state of a disease is used instead of death. Indirect measures include the Health Utilities Index and the European Group Quality of Life 5; both are validated for use in adolescents 12 years and older [32,33]. The problem with these measures is that the predetermined utility weights currently used for these questionnaires are based on adult preferences, which may compromise their use for adolescents [29]. However, an Australian group evaluated another measure, the Assessment of Quality of Life instrument. The authors conducted a recalibration study and were able to derive utility weights specifically for adolescents [34]. The study included adolescents from 4 different countries and used algorithms and multiplicative models to develop age- and country-specific utility weights [34].

Nonadherence to the recommended treatment is a widespread problem in pediatric CHCs. The increasing prevalence of CHCs coupled with management problems in pediatric populations present a barrier to optimal health. Self-management skills in adolescents and young adults are critical to maintain optimal adherence to their chronic medications, especially when they transition from pediatric to adult facilities with more expectations of self-care. Adolescent-centered interventions are needed to optimize their adherence to prescribed medication across CHCs, support the development of self-management skills, and enhance intervention uptake and long-term engagement while transitioning to self-care.

There has been a growing interest in the use of technology to improve medication adherence and self-management skills in the last few years. Similarly, there has been increased interest in the use of portable and easily accessible technology-based approaches to address health-related problems with an overall acceptability and feasibility for different health conditions. Among adults, evidence for the efficacy of text messaging to support medication adherence exists [35,36] and the evidence among adolescents with chronic conditions is emerging [15-17]. While results of these studies are promising, they suggest that additional adherence intervention development is needed and should be tested with more rigorous designs and across a broader range of chronic conditions.

Despite the growing evidence of the efficacy of text-messaging and smartphone app interventions in improving medication adherence in adults with CHCs [35-38], to date there have been no formal economic evaluations of these interventions, and their cost effectiveness remains unclear [38]. However, there is evidence to support the cost effectiveness of different technology-based interventions to promote behavior change among adults, such as smoking cessation [39-41].

The majority of smartphone app initiatives have been pilot studies and the data generated from these studies are limited. In addition to efficacy and effectiveness data, economic evaluation is warranted. The cost to develop and maintain each intervention could be a barrier to the use of these interventions on a broader scale. Additionally, there is variability in patient access to preferred technologies. Formal economic evaluation of various interventions will help health care authorities determine whether the investment required to develop, maintain, and disseminate these interventions is worth the broader benefit, or lack thereof, experienced by patients with chronic conditions. Given the emerging evidence in the field of eHealth, future economic evaluations could consider broader inclusion criteria for different technology-based interventions.

### Conclusion

In conclusion, we found no evidence to support the cost effectiveness of technology-based text-messaging and smartphone app interventions. The effect of such technology tools on health care costs in adolescents with CHCs can be beyond medication adherence. Technology-based interventions can facilitate increased operating efficiencies (eg, fewer missed clinic appointments), increased access to high-quality health services (eg, timely referrals to other services for consultations and self-management tools), and potential labor cost savings. Economic evaluation of technology-based interventions can contribute to a better and more evidence-based assessment of the scalability, sustainability, and benefits of broader investment of such technology tools and may raise considerations for third-party reimbursement should interventions prove to be effective in improving health outcomes in this population.

## Appendix

Multimedia Appendix 1 Database search strategies.

## Abbreviations

CENTRAL: Cochrane Central Register of Controlled Trials
CHC: chronic health conditions
CINAHL: Cumulative Index to Nursing and Allied Health Literature
CRD: Center for Review and Dissemination
DOT: directly observed therapy
GRADE: Grades of Recommendation, Assessment, Development, and Evaluation
HRQOL: health-related quality of life
ICER: incremental cost-effectiveness ratio
PRISMA: Preferred Reporting Items for Systematic Reviews and Meta-Analyses
QALY: quality adjusted life years
